# Understanding attention deficit disorder: a neuroscience prospective

**DOI:** 10.3325/cmj.2014.55.174

**Published:** 2014-04

**Authors:** Lukasz M. Konopka

**Affiliations:** Department of Psychiatry, Loyola Medical Center, Maywood, IL and Yellowbrick Consultation and Treatment Center, Evanston, IL, USA

Today’s ready access to electronic devices and the internet often substitute for social interaction. Such situation, if it starts in early childhood, in certain vulnerable individuals, who crave immediate rewards, can decrease the ability to maintain prolonged attention (1) and tolerate delayed gratification, thus reinforcing future addictive behaviors (2). Once established, these behaviors, such as attention deficit hyperactivity disorder (ADHD), may persist into adulthood. ADHD is one of the most common reasons for referring children to mental health facilities. Generally, parents seek professional help for their children after the school suggests that their child has difficulties. These difficulties may be related to academic performance, behavior, or both. Research data support the idea that children with attention deficit disorder often come from families with attention deficit in one or both parents (3). Very recent data indicate a significant genetic predisposition for this disorder. Seventy percent of children who have this disorder will have the disorder as teenagers, and about 40%-60% will still have the disorder as adults, and although genetic studies have not isolated a gene for ADHD, there may be several genes contributing to the vulnerability for developing this disorder. For example, twin studies have shown a significant heritability for ADHD (4) as high as 76%. In addition, the parents and siblings of children with ADHD have an ADHD diagnostic probability 4-5 times higher than the general population, and boys are more vulnerable than girls (5). Based on these data, it might appear that ADHD is easily diagnosed and, thus, simple to treat, but we still must question the methods and criteria used for diagnosing ADHD. Currently, clinical interviews and collateral histories from parents and teachers drive the standards. Only occasionally do we use objective assessments, such as continuous performance tasks and neuropsychological assessments, to evaluate whether or not a child can sustain attention, and whether their deficits lie in either or both auditory and visual domains. Various factors may play a role in sustaining attention. These may include: motivation, concurrent anxiety, lack of sleep, low blood glucose, medication, and family collaboration (6). Clinicians consider and evaluate three major symptom groups for diagnosing ADHD: inattention, hyperactivity, and impulsivity. Each category describes particular symptoms; for example, inattention may involve an inability to finish tasks, organize, and sustain efforts as well as forgetfulness and distractibility. Hyperactivity is defined as being fidgety, inability to sit still, and motoric hyperactivity – excessive running, climbing, and moving. Impulsivity involves excessive talking, answering without thinking, inability to wait one’s turn, interrupting, and so on. Both the DSM IV and the ICD 10 provide guidelines for the frequency and duration of the symptoms in these diagnostic categories, and both generally agree on the necessary number of symptoms before someone receives the diagnosis of ADHD. Nevertheless, it should be noted that these symptoms may be defined and scored by individuals that are untrained in this process. Therefore, we need to question the reliability and validity of such unidimensional data.

On the other hand, neuroscientists focus primarily on the relationship between the symptoms and brain structures such as the dorsal lateral prefrontal cortex, ventral lateral prefrontal cortex, insula, anterior cingulate, and default networks and functions linked to neurotransmitter systems (7-10). They generally agree that ADHD patients primarily struggle with improper utilization of the neurotransmitter dopamine, and, to a lesser extent, norepinephrine (11,12). Dopamine significantly affects numerous behaviors, including movement modulation, cognition, mood, and attention. In ADHD cases primarily involving dopamine, clinical medication interventions can increase dopamine’s availability, significantly improve continuous task performance, decrease hyperactivity, and increase behavior management in school. Although methylphenidate is considered the first-line medication for attention deficit disorder hyperactivity in children and adults, many children are unresponsive to this medication. We should ask ourselves why. Recent neuroscientific studies describe three types of attentional networks: ventral, dorsal, and default executive control. Although these network functions overlap (13), if any network deregulates, it will contribute to some clinically observable aspects of ADHD. Therefore, if we learn to understand the identified risk factors that lead to the clinical development of this disorder, ie, *in utero* drug exposure, low birth weight, premature delivery, endocrine deregulation, early developmental neglect, environmental toxins, head trauma, and genetic vulnerability, we can understand the vulnerability and deregulation of these networks (6).

Quantitative EEG has helped define ADHD’s physiological underpinnings by clearly identifying electrical patterns such as increased frontal slowing and excessive frontal theta (14) that correlate with inability to sustain attention (15). Based on this information, the Food and Drug Administration recently approved qEEG as an adjunct assessment tool for the diagnosis of ADHD (16). From objective qEEG measures, new data suggest that varying electrical patterns may fit the definition of childhood inattention, in the ADHD category (13), and point to a heterogeneous ADHD population (17). The challenge lies in understanding how different patterns respond to different treatments.

Then we should ask what happens to the child as they become an adult? To answer this question, we need to understand the adult presentation of attention deficit disorder (18,19). Generally, although the specific ADHD symptoms for children and adolescents differ from adults, we can observe and define similar behaviors. In adults, inattention and impulsivity are less evident; rather their symptoms manifest as poor concentration, distractibility, an inability to finish tasks, and poor planning, which, in turn, may become an inability to maintain employment (20), and because these individuals are often drawn to fast-paced, highly stimulating work with immediate rewards, they may leave a job out of boredom and prefer employment in the stock market, sales, emergency department, or creative jobs that give them ample freedom (21,22). In addition, these individuals often engage in self-destructive behaviors such as substance abuse, gambling, and other immediately rewarding behaviors. Simply stated, they have difficulty delaying gratification. We can effectively treat these adults, just as we would treat a child, by combining medications, such as dopamine and, perhaps, norepinephrine that target their symptoms, with behavioral and psychological interventions (18).

Nevertheless, our current data support the idea that, in many cases, ADHD persists throughout one’s life: when untreated, ADHD can have significant psychiatric comorbidities that include anxiety, depression, substance abuse, conduct disorder, and bipolar disorder (23). Therefore, to effectively treat these individuals, we must establish a clear diagnosis using multi-dimensional diagnostic evaluations that address patients from bio-psychosocial and spiritual points of view. Moreover, comprehensive, early developmental, familial, functional, neuropsychological, and neurophysiological assessments are vital. The converging data from these various approaches will provide a better understanding of how the patients perceive their environment, and how they respond to stress, solve problems, and react to rewards or punishment. Afterwards, when considering medication therapies, we can choose according to patient-specific pharmaco-dynamic and pharmo-kinetic data. Later, to further tailor the patient’s medication paradigm, we can use qEEG acute medication challenge studies and neuro-behavioral assessments (24,25). Recent data show that methylphenidate and atomoxetine differ in their effects; methylphenidate decreases brain noise by increasing signal-to-noise ratio and enhances overall performance, while atomoxetine increases specific performance and the salience of task-specific signals (26,27). This pharmaco-therapeutic approach may enhance medication effects and avoid prolonged clinical trials that can last for days and weeks with significant hardship for the patients and their families.
